# What is known about the care and support provided for an ageing population with lived experience of chronic viral hepatitis as they near end‐of‐life: A scoping review

**DOI:** 10.1111/hsc.14066

**Published:** 2022-10-19

**Authors:** Kerryn Drysdale, Jake Rance, Elena Cama, Carla Treloar, Limin Mao

**Affiliations:** ^1^ Centre for Social Research in Health UNSW Sydney Sydney Australia

**Keywords:** end‐of‐life, hepatitis B, hepatitis C, palliative care, scoping review

## Abstract

Ageing with a chronic hepatitis B (HBV) or hepatitis C (HCV) infection is an emerging public health priority. For people living with chronic viral hepatitis, their disease progression into old age is both underpinned by their existing blood borne virus and the potential emergence of other infectious and non‐infectious conditions. These twinned pathways bring additional challenges to the care and support for people as they near end of life. This scoping review sought to examine what is known about the experiences of the end‐of‐life phase of an increasing population ageing with HBV and HCV in studies conducted in high‐income settings and published in peer reviewed literature (2010–2021). In interpreting this literature, we found that challenges in determining the end‐of life phase for people with lived experience of HBV or HCV are exacerbated by the conflation of aetiologies into a singular diagnosis of end‐stage liver disease. Studies overwhelmingly reported the clinical aspects of end‐of‐life care (i.e. prognosis assessment and symptom management) with less attention paid to educative aspects (i.e. advance care directives and surrogate decision makers, discussion of treatment options and determining goals of care). Psychosocial interventions (i.e. quality of life beyond symptom management, including emotional/spiritual support and family and bereavement support) received limited attention in the literature, though there was some recognition that psychosocial interventions should be part of end‐of‐life care provision. Given the focus on the prominent disease presentation of liver cirrhosis and/or end‐stage liver disease, the social and cultural dimensions of these infections have received less attention in the literature on end‐of‐life in the context of chronic viral hepatitis.

**What is known about this topic?**

- Increasing commitment to the global elimination of viral hepatitis has meant that people are now ageing with lived experience of hepatitis B and/or hepatitis C.
- People living with chronic hepatitis B and/or hepatitis C represent a distinct population associated with higher rates of age‐related co‐morbidities and other specific care needs.
- There is currently little research existing that addresses the intersection of ageing, chronic illness and end‐of‐life care in the context of chronic viral hepatitis.

**What does this paper add to this topic?**

- Synthesising insights into determining the end‐of‐life phase for people with lived experience of hepatitis B and/or hepatitis C, as well as their care and support needs at this stage of life, confirms the limited range of evidence available.
- Critical to interpreting this evidence is the question of how viral hepatitis is understood in relation to the end‐of‐life phase given the focus on the prominent disease presentation of liver cirrhosis and/or end‐stage liver disease.
- An emphasis on clinical and educative aspects of end‐of‐life care meant that psychosocial dimensions received less attention in the literature, and the social and cultural dimensions of ageing and dying with hepatitis B and/or hepatitis C were obscured.

## 1 INTRODUCTION

Ageing with a chronic hepatitis B (HBV) or hepatitis C (HCV) infection is an emerging public health priority as people living with these inflections represent a range of specialised health needs among Australia's rapidly ageing population. In particular, the increasing life expectancy of these affected populations warrants specific attention to the effects of co‐morbidities and poly‐pharmacies that may contribute to or compound ageing as they near end of life (Card et al., 2020; Heron et al., 2019; Siefried et al., 2018). At the same time, behavioural, social and structural factors may impede equity in health outcomes at this life phase (Mao et al., 2019). While HBV and HCV are distinct diseases and should not be conflated in clinical contexts, people with lived experience of HBV or HCV can be united by their chronic condition, long asymptomatic periods of infection, long‐term effects on the liver and the stigma associated with the diseases. Accordingly, we sought to determine how end‐of‐life care and support is provided for an increasing population of people ageing with chronic HBV and/or HCV; that is, the care given to people who have stopped treatment to cure or control their disease as they are considered to be at the end of their life course (which can include physical, emotional, social and spiritual support for patients and their families). Limited existing research and the pressing need for stronger evidence for this ageing population warrants the examination of HBV and HCV concurrently.

### 1.1 Background

In 2016, the World Health Organization drew worldwide attention to the issue of viral hepatitis with the publication of its inaugural global health strategy that set ambitious targets for the elimination of viral hepatitis as a major public health threat by 2030 (World Health Organization, 2016). Effective vaccination, improved treatment and cure (in the case of HCV) mean that living and dying in older age with lived experience of HCV or HBV is now possible—and probable—for many (El‐Serag & Kramer, 2008; Wong et al., 2020). As such, people who live with the effects of chronic HBV or HCV represent an emerging subset of an ageing population. While experiences of ageing are challenging for all (The Academy of Medical Sciences, 2018; Meagher et al., 2019), people living with chronic HBV or HCV represent a distinct population associated with higher rates of age‐related co‐morbidities and other specific care needs (Mao et al., 2019; Richmond & Wallace, 2014; Ridruejo & Silva, 2012). Outside of clinical or physical symptomatology, older people living in marginalised social circumstances also experience fewer opportunities for end‐of‐life support due to social or economic deprivation, stigmatisation, and/or poor health (Ellis et al., 2016; Hughes & Cartwright, 2015). Yet, little research exists that addresses the intersection of ageing, chronic illness and end‐of‐life care in the context of chronic viral hepatitis and the small number of studies that have done so have concentrated on specific groups that reflects their different needs, such people who inject drugs, migrant communities and people experiencing homelessness (McNeil & Guirguis‐Younger, 2012; Sumalinog et al., 2017; Taylor et al., 2006).

Globally, it is estimated that approximately 296 million people were chronically infected with HBV in 2019 (World Health Organization, 2021). Only 30.4 million people (10.5% of all people estimated to be living with HBV) were aware of their infection and less than a quarter (22%) of the people diagnosed were on treatment in 2019 (World Health Organization, 2021). The absolute number of infected individuals continues to grow in line with the global population, with 1.5 million new infections each year (World Health Organization, 2021). Clinical management, including regular monitoring and pharmaceutical treatment effectively reduces the risk of mortality resulting from the infection (Lok & McMahon, 2009). Additionally, the introduction of neonatal vaccination programs has considerably reduced the incidence and prevalence of HBV infection in several areas of high endemicity, and in Australia, where the authorship team are based, the Australian National Immunisation Program now provides HBV vaccination at birth and during infancy. While the 95% overall National Strategy target for infant HBV immunisation was reached in 2020, it is estimated that 27% of the people living with HBV in Australia remain undiagnosed (MacLachlan et al., 2020). Of the estimated 222,559 people in Australia living with HBV in 2020, the majority (61%) of them were born overseas (MacLachlan et al., 2020), therefore incidence cannot be prevented through local vaccination initiatives alone. As a result, the prevalence of HBV‐related disease among the Australian population is estimated to increase in the future.

An estimated 71 million people are living with chronic HCV infections around the world (World Health Organization, 2021), including an estimated 199,230 Australians (Kirby Institute, 2018). While WHO estimates that 290,000 people died from HCV‐related causes in 2019 (World Health Organization, 2021), new data also show that 9.4 million people are receiving treatment for chronic infections—a nearly 10‐fold increase from 2015 (World Health Organization, 2021). Unlike HBV, HCV treatments affording the possibility of cure have been available for several decades. Until recently, however, the only option has been interferon‐based therapy, a notoriously long and arduous regime with limited success rates (Hellard et al., 2009). The treatment landscape was dramatically reconfigured with the advent of new direct‐acting antivirals (DAAs) in 2014. Described as ‘one of the great advances in clinical medicine in recent decades’ (Dore & Hajarizadeh, 2018, p. 269), DAAs offer simpler, shorter adherence regimens, markedly reduced side effects and cure rates of near 100% (Banerjee & Reddy, 2016; Dore & Feld, 2015). Yet, the absence of an HCV vaccine means that behavioural interventions (such as the use of sterile injecting equipment) remain the chief form of prevention for both primary infections and reinfections. Limited empirical evidence exists for the effectiveness of HCV treatment‐as‐prevention (i.e. treatment as a tool for limiting the spread of an infection in epidemics in a particular setting; see Hajarizadeh et al., 2016). In Australia, early trials show a significant reduction in HCV incidence for people who inject drugs in carceral settings (Hajarizadeh et al., 2021), but scaleup outside of these settings has been limited to date. Improving rates of testing and diagnosis are also crucial, with only 15.2 million out of the estimated 71 million people living with HCV worldwide aware of their serostatus (World Health Organization, 2021). Due to the slow progression of the virus, the more severe consequences of HCV typically manifest 30 years post infection. Low testing rates and the often‐asymptomatic nature of acute infections mean that people can live for years with undetected infection and the possibility of developing long‐term liver damage before diagnosis and treatment (World Health Organization, 2021). As a result, people who were infected with HCV in their 20s are now at heightened risk of liver damage by the time they reach their 50s (Shepard et al., 2005). Moreover, despite the clear benefits of DAA therapy through HCV infection cure, potential longer‐term clinical implications are less known (Alavi et al., 2019). While successful courses of DAA treatments among HCV‐affected people have resulted in lower mortality among younger populations, ongoing risk of liver‐related diseases are still present among older populations, especially for those where there is a lengthy delay between infection and treatment.

What constitutes ‘end of life’ in the context of ageing for these cohorts is difficult to determine. On the one hand, there is a lack of clarity around accelerated ageing linked to hepatitis‐related morbidities, given differences between symptomatolgy, iatrogenic effects and other comorbidities. On the other hand, an individual's hepatitis status—and their care and support needs related to that infection—can be obscured by other more prominent disease profiles as people near the end of life. Moreover, there is a need to understand whether age is an appropriate proxy for ‘severity of liver disease’ (Richmond & Wallace, 2014) or whether there are specific age‐related issues associated with HBV and HCV outside of that disease. Generally, end‐of‐life detertminations and palliative care clinical referrals are based on disease progressions that have definitive markers (e.g. a quick or a prolonged, but overall consistent, decline of physical function and quality of life where treatment options are no longer life sustaining) (Amblàs‐Novellas et al., 2016; Lunney et al., 2003). HBV and HCV viral infection may indeed confound this determination, given the curative potential of treatment and/or liver transplant. Moreover, while the benefits of timely referral to palliative care are well‐established in other disease contexts (Friedman et al., 2002; Hui et al., 2018; Temel et al., 2010), people with end‐stage liver disease experience low rates of referral to specialist palliative care, and those who are referred are often delayed or very late (Kimbell & Murray, 2015; Low et al., 2018; Peng et al., 2019; Sumalinog et al., 2017; Valery et al., 2015). Collectively, these factors highlight considerable challenges in meeting the supportive and end‐of‐life care needs in the context of ageing with HBV and HCV.

## 2 METHODS

A scoping review methodology was determined to be the most appropriate method for responding to the research question: how is end‐of‐life care and support provided for an increasing cohort of people ageing with lived experience of HBV and HCV? (See Table 1). A scoping review provides a transparent, rigorous and structured process for exploring the literature in order to map the breadth of existing research—and to identify knowledge and research gaps—on a specific topic of interest, especially where there is limited published research (Arksey & O'Malley, 2005). Unlike narrative reviews, scoping reviews require ‘analytical reinterpretation’ of the published study findings, which is valuable for assessing the contribution of the research that is available (Levac et al., 2010). Compared to systematic reviews, scoping reviews do not focus on evaluating the methodological quality of the studies being appraised, which means there is greater flexibility in looking across a diverse range of study types (Arksey & O'Malley, 2005).

**TABLE 1:** Outline of steps undertaken for the scoping review

Stage 1—identifying the research question	*Purpose of review*: To determine what is known about end‐of‐life care and support for an ageing cohort of people with lived experience of chronic viral hepatitis *Research question*: How is end‐of‐life care and support provided for an increasing cohort of people ageing with lived experience of HBV and HCV?
Stage 2—identifying relevant studies	*Databases*: ProQuest, Scopus, Pub Med, Web of Science, Cochrane *Reference checks*: Google Scholar *Language*: English only *Type of publication*: journal article, book, book chapter *Search terms*: A three‐tier search term system whereby the first search tier (populations) was combined with each combination in the second and third tiers (in article title, abstract, key words). T1 (populations): Hepatitis OR HBV OR HCV OR liver cancer OR liver disease OR liver failure OR hepatal OR hepatology OR hepatic OR cirrhosis OR hepatocellular carcinoma OR hepatoma OR PWID OR “people who inject drugs”. T2 (subject): end‐of‐life OR end‐stage OR dying OR death OR terminally ill OR terminal* T3 (subject): palliative care OR hospice care OR hospice OR supportive oncology OR advance care planning
Stage 3—study selection	*Exclusion criteria*: Publication not in English; not relevant to research question; studies focusing on young people's mortality; clinical protocols and practitioner guidelines; articles on palliative care not in the context of the target population; articles on liver that do not explicitly refer to HBV and/or HCV; RCTs for clinical trials/drug interventions; letters to the editor or commentary, reviews not containing original research; media articles (magazines, newspapers), theses and conference papers; peer‐reviewed publications published before 1/1/2010. *Inclusion criteria*: English language; relevant to research question, inclusion of ‘hepat*’ in full article text, peer‐reviewed publications containing original research published after 1/1/2010.
Stage 4—charting the data	All included references were charted in Excel where relevant information was synthesised and interpreted by sifting, charting and sorting according to key issues and themes. A ‘descriptive‐analytical’ method was used, which involves applying an analytical framework to the literature and collating information on each. The list of included references were exported into the ‘data charting form’, and a coding framework developed to characterise key features of each.
Stage 5—reporting the results	All results were collated to provide an overview of the material reviewed, categorised into themes that responded to the following questions: How is end of life determined in the context of ageing with lived experience of chronic viral hepatitis? How is care and support at end‐of‐life provided for people with lived experience of chronic viral hepatitis?
Stage 6—consultation exercise	Informal consultations took place with three expert key informants from relevant organisations or with relevant expertise across July and September 2021.

**FIGURE 1:**
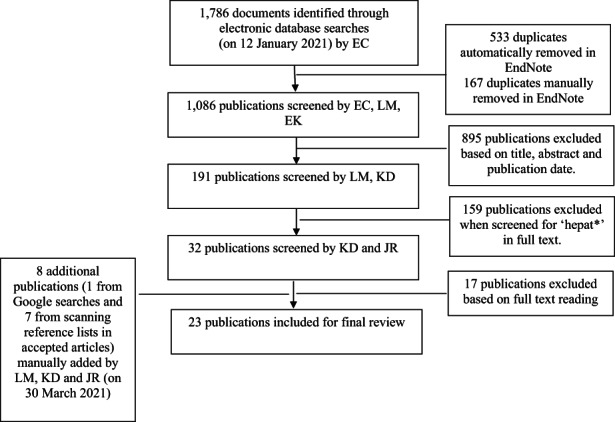
Flowchart of selection process

This review scoped peer‐reviewed academic literature published in English from 1 January 2010 to 12 January 2021. This date range was selected to reflect the significant changes in the relevant fields of viral hepatitis, palliative and/or end‐of‐life care; that is, to capture changes following the introduction of DAAs for HCV infection alongside palliative medicine becoming established as an area of speciality. Relevant literature was identified by searching key databases with consistent search parameters and using EndNote as a data management tool (see Table 1 for a list of databases and search parameters and Figure 1 for a chart of the selection process). Keywords aimed to capture all intersecting research concerning the disease profile (HBV, HCV and their comorbidities and mortalities), phase of life (ageing, dying and end‐of‐life) and treatment type (palliative and supportive care, advance care planning and other non‐clinical and non‐curative care). Editorials, letters to the editor, reviews, and/or commentaries with no original research were excluded, as were publications where their main content was clinical protocols or practitioner guidelines, or articles on palliative care not in the context of the target population. Articles that explored experiences of liver cirrhosis associated with HBV or HCV where findings also referenced palliative care and/or advance care planning in both acute and non‐acute settings were included.

At each stage in the overall process, screening was undertaken independently by at least two of the authors, and any disparities over inclusion or exclusion were resolved by a further independent screening by another author until consensus was reached. As our focus was on an ageing population with living experience of HBV and/or HCV, a separate screening of selected peer‐reviewed literature was conducted to ensure only literature that mentioned hepatitis in the full article text were included (i.e. excluding articles focussing only on liver disease with no consideration of its aetiology). Twenty‐three publications were identified as eligible for inclusion in the final analysis, which is well aligned with scoping review methodology (Arksey & O'Malley, 2005). The research team read each article closely and iteratively, then convened to discuss initial impressions. Through this process, research sub‐questions were then formulated to guide the analysis: how is end‐of‐life determined in the literature and based on this determination, what care and support was provided at end‐of life? Consultations with experts working in the field of viral hepatitis were conducted to seek their guidance on the scope and interpretation of findings, in line with the recommendations of scoping review methodology (Levac et al., 2010).

## 3 RESULTS

The search strategy generated 1094 unique citations of which 23 articles met the inclusion criteria (see Table 2). The majority of articles listed HBV and/or HCV in the context of the aetiology of liver disease; that is, presented only in terms of participant/patient demographics with no additional analysis or commentary. HCV was most frequently listed in the scoped literature (and the only listed hepatitis‐related aetiology in 11 of the articles reviewed), noted as either separated from or combined with ‘alcohol’ or ‘alcoholic liver disease’. A further seven articles listed both HCV and HBV in their presentation of patient characteristics, and smaller set of two articles did not distinguish between HCV and HBV in their listing of ‘viral hepatitis’. Fukui et al. (2017) specifically excluded patients with nonspecific hepatitis and HBV in their analysis (retaining only HCV among other non‐hepatitis disease aetiologies), and Tsai et al. (2018) noted that the non‐stratification of disease aetiology in their mortality prediction was a limitation of their study (see also Hansen, Leo, et al., 2015). Given the overall lack of differentiation over how ageing and associated morbidities intersect with clinical markers that might indicate the need for end‐of‐life care, we organised our analysis into two broad domains that responded to the research sub‐questions posed, the relevance of which was confirmed by consultations with experts. These two domains explore the challenges in distinguishing both the end‐of‐life phase and the care that is provided at this point in an individual's life course.

**TABLE 2:** Final selection of articles

Citation	Study type	Study country	Study population	Study site	End‐of‐life phase
Baumann et al. (2015)	Quantitative—Survey	United States	End‐stage liver disease patients awaiting liver transplantation (*n* = 50); mean age 58.9	Liver transplant centre	Palliative care intervention where eligibility was determined by broader site enrolment.
Brown et al. (2016)	Quantitative—Retrospective	United States	Hospitalised end‐stage liver disease patients (*n* = 22,311); mean age 70.9	Random selection of national Medicare records	Liver clinic inpatients, or hospice or hospital admissions with diagnoses of chronic liver disease, cirrhosis and/or haptic decompensation.
Carbonneau et al. (2018)	Qualitative—Interview and focus group	Canada	Patients with cirrhosis (*n* = 17); age range 40 to 79	Cirrhosis care clinic	Not defined.
Chen et al. (2021)	Quantitative—Retrospective	Australia	Patients with chronic liver disease (*n* = 116); mean age 59	Admissions to a tertiary referral centre.	Model for End‐Stage Liver Disease (MELD) and/or Child–Turcotte–Pugh (CTP) scores as a measurement of liver decline
Ebenau et al. (2019)	Qualitative—Interview	The Netherlands	Patients with ‘substance use disorder’ receiving palliative care (*n* = 9); mean age 61; also include ‘proxies’ and ‘healthcare professionals’ perspectives	Various	Not defined.
Fukui et al. (2017)	Quantitative—Retrospective	United States	Elderly patients with chronic liver disease (*n* = 2179); mean age 70.43	Random selection of national Medicare records	Liver clinic inpatients, or hospice or hospital admissions with diagnoses of chronic liver disease, cirrhosis and/or haptic decompensation.
Gray‐Renfrew et al. (2020)	Qualitative—Interview	United Kingdom	Patients with advanced liver disease (*n* = 15); age range 35 to 84	Inpatient liver clinic	Liver clinic inpatients, or hospice or hospital admissions with diagnoses of chronic liver disease, cirrhosis and/or haptic decompensation.
Hansen et al. (2014)	Quantitative—Prospective longitudinal	United States	Patients with terminal hepatocellular carcinoma (*n* = 20); mean age 59	2 hepatology clinics	Model for End‐Stage Liver Disease (MELD) and/or Child–Turcotte–Pugh (CTP) scores as a measurement of liver decline; level of pain burden as indicative of reduced quality of life
Hansen, Leo, et al. (2015)	Quantitative—Prospective descriptive	United States	Patients with end‐stage liver disease (*n* = 20); mean age 59	Outpatient liver clinic	Model for End‐Stage Liver Disease (MELD) and/or Child–Turcotte–Pugh (CTP) scores as a measurement of liver decline; level of pain burden as indicative of reduced quality of life
Hansen, Rosenkranz, et al. (2015)	Qualitative—Longitudinal descriptive	United States	Patients with end‐stage liver disease (*n* = 20); mean age 61.5	Outpatient liver clinic	A diagnosis of hepatocellular carcinoma beyond the Milan criteria for liver transplant
Kimbell et al. (2015)	Qualitative—interview	United Kingdom	Patients with advanced liver disease (*n* = 15); age range 35–84; also includes ‘lay carers’ and ‘healthcare professionals’ perspectives	Inpatient liver unit	Liver clinic inpatients, or hospice or hospital admissions with diagnoses of chronic liver disease, cirrhosis and/or haptic decompensation.
Lamba et al. (2012)	Mixed methods	United States	Liver Transplant candidates (*n* = 79); mean age 51; also includes ‘family’ perspectives	Liver transplant centre	Palliative care intervention where eligibility was determined by broader site enrolment.
Low et al. (2017)	Mixed methods	United Kingdom	Patients with advanced chronic liver disease in the last year of life (*n* = 30); mean age 58; also includes ‘healthcare professionals’ perspectives	Liver transplant centre	Palliative care intervention where eligibility was determined by broader site enrolment.
Najafian et al. (2019)	Quantitative—Retrospective	United States	Patients with cirrhosis (*n* = 18); mean age 56.	Transitional care (hospital to hospice) liver clinic	Liver clinic inpatients, or hospice or hospital admissions with diagnoses of chronic liver disease, cirrhosis and/or haptic decompensation.
O'Leary et al. (2020)	Quantitative—Prospective	United States	Non‐electively hospitalised patients with cirrhosis (*n* = 2718); mean age 58.96	14 tertiary‐care hepatology centres	Liver clinic inpatients, or hospice or hospital admissions with diagnoses of chronic liver disease, cirrhosis and/or haptic decompensation.
Patel et al. (2020)	Quantitative—Retrospective	United States	Veterans with end‐stage liver disease (*n* = 12); mean age 61	Veterans healthcare service	Model for End‐Stage Liver Disease (MELD) and/or Child–Turcotte–Pugh (CTP) scores as a measurement of liver decline
Podymow et al. (2006)	Quantitative—Retrospective	Canada	Terminally ill people experiencing homelessness (*n* = 28); mean age 49	Shelter‐based palliative care service	Liver clinic inpatients, or hospice or hospital admissions with diagnoses of chronic liver disease, cirrhosis and/or haptic decompensation.
Poonja et al. (2014)	Quantitative—Retrospective	Canada	Patients with cirrhosis and denied liver transplants (*n* = 102); mean age 56	Liver transplant centre	A diagnosis of hepatocellular carcinoma beyond the Milan criteria for liver transplant
Shinall et al. (2019)	Mixed methods	United States	Patients with end‐stage liver disease (*n* = 63); mean age 57.95 (control) and 58.28 (intervention)	Admissions to hepatology service at referral centre	Palliative care intervention where eligibility was determined by broader site enrolment.
Sprange et al. (2020)	Quantitative—Prospective	Canada	Patients with cirrhosis (*n* = 97); mean age 61.8	2 cirrhosis clinics	Liver clinic inpatients, or hospice or hospital admissions with diagnoses of chronic liver disease, cirrhosis and/or haptic decompensation.
Tsai et al. (2018)	Quantitative—Retrospective	Taiwan	Patients with non‐ cancer‐related end‐stage liver disease (*n* = 4080); mean age 53 (non‐mortality) and 50 (mortality)	Admissions and outpatients of hospital	Liver clinic inpatients, or hospice or hospital admissions with diagnoses of chronic liver disease, cirrhosis and/or haptic decompensation.
Valery et al., (2015)	Mixed methods	Australia	Patients diagnosed with cirrhosis (*n* = 33); mean age 58; also includes ‘healthcare professionals’ perspectives	Tertiary healthcare facility	Liver clinic inpatients, or hospice or hospital admissions with diagnoses of chronic liver disease, cirrhosis and/or haptic decompensation.
Wang et al. (2021)	Quantitative—Retrospective	United States	Liver Transplant Candidates (*n* = 170); mean age 58	Liver transplant centre	Liver clinic inpatients, or hospice or hospital admissions with diagnoses of chronic liver disease, cirrhosis and/or haptic decompensation.

### 3.1 Challenges in determining end‐of‐life phase in the context of ageing with chronic viral hepatitis

The identification of the need for end‐of‐life care is complex as trajectories from ageing to dying can encompass a range of co‐morbidities and disease presentations. The majority of articles determined disease progression to end‐stage or advanced liver disease (where clinical interventions to prolong life were likely to be limited) as an indicator that a patient was nearing the end of life. Understanding the needs of people with advanced liver disease was further complicated by the possibility of curative liver transplant. Studies that specifically excluded patients on liver transplant waiting lists from an end‐of‐life determination (*n* = 8) did so on the basis that a transplant that would cure current liver disease (Fukui et al., 2017; Gray‐Renfrew et al., 2020; Hansen et al., 2014; Hansen, Leo, et al., 2015; Hansen, Rosenkranz, et al., 2015; Kimbell et al., 2015; Najafian et al., 2019; Poonja et al., 2014). Conversely, studies that specifically included patients on liver transplant waiting lists (*n* = 6) highlighted the unpredictable progression of liver disease, the expectation of worsening somatic and psychological symptoms, lengthy waiting lists, and the trajectory of decline in quality of life (Baumann et al., 2015; Carbonneau et al., 2018; Lamba et al., 2012; Patel et al., 2020; Shinall et al., 2019; Wang et al., 2021). Tsai et al. (2018) developed a more precise prognostic model to define an end‐of‐life trajectory for patients with non‐cancer‐related end‐stage liver disease because of the fluctuation in deterioration that patients experience. All research studies excluded people with cognitive impairment that inhibited informed consent, though prospective and retrospective cohort studies noted the presence of hepatic encephalopathy (impaired brain function as a result of advanced liver disease) as a complication in navigating healthcare systems and engaging in advance care planning processes (Najafian et al., 2019; O'Leary et al., 2020; Patel et al., 2020; Wang et al., 2021).

How the end‐of‐life phase was determined (see Table 2) had implications for the introduction of end‐of‐life care, often reflected in eligibility criteria for specialist palliative care. Chen et al. (2021) defined eligibility for specialist palliative care through the Supportive and Palliative Care Indicators Tool, a combination of general indicators of deteriorating health and clinical indicators of advanced liver disease, to identify patients who would likely benefit from early palliative referral. Using these indicators, they found that only a fifth of patients who met eligibility (25/116) were referred to palliative care. Yet, findings show disparity between who is referred, with lower referral rates for patients with HBV‐ and/or HCV‐related, along with alcohol‐related, liver disease as compared with non‐alcoholic steatohepatitis (noting that HCV and HBV were combined as ‘viral hepatitis’ in the results) (Chen et al., 2021). Poonja et al. (2014) evaluated cirrhotic patients who were deemed palliative, which they defined as those for whom curative potential for liver disease was exhausted (i.e. declined or delisted for liver transplantation). They found 11% were referred to palliative care despite large prevalence of somatic and psychological symptomatology and high rates of mortality among this cohort. While they found that older patients (over 60 years of age) were more likely to be assessed by palliative care teams than younger patients, they concluded that specialist palliative care was underutilised for all patients in their study. Low et al. (2017) analysed 30 purposefully sampled patient records with cirrhosis in the last year of life to describe a highly symptomatic population. Despite increasing frequency of in‐patient admissions for most people in the last 3 months of life, referrals to specialist palliative care took place a median of 5 days before death, with 40% of records referring to active treatment up until death. Moreover, the authors noted that when discussions around prognosis and care preferences outside of specialist palliative care occurred, these discussions were conducted very late (approximately 1 month before death). In a prospective study of veterans with end‐stage liver disease, Patel et al. (2020) found that while palliative care provision was provided to just over half of their cohort despite high anticipated mortality, palliative care referral was positively associated with highest overall quality indicators of supportive care and information care planning, which indicates that there are benefits even with late or low referrals.

Articles also evaluated referrals to hospice (as a proxy for palliative care) among patients with end‐stage liver disease. Brown et al. (2016) compared hospice enrolment rates between three groups: patients with end‐stage liver disease without a concurrent cancer diagnosis, patients with end‐stage liver disease with a concurrent cancer diagnosis and patients with heart failure (the latter as a reference group). The authors found that the confounding association of liver disease with cancer resulted in higher rates of discharge to hospice, comparable to those patients with heart failure. However, despite similar mortality and readmission rates between patients with end‐stage liver disease and patients with heart failure, 3.9% of patients with end‐stage liver disease without a corresponding diagnosis of cancer were discharged to hospice compared to 9% of patients with cancer. Similarly, O'Leary et al. (2020) prospectively enrolled hospitalised patients with cirrhosis to determine predictors of hospice utilisation (noting that data on the frequency of palliative care consultations was not available). They found that the 5% of their sample who were discharged to hospice (the remainder died in hospital or were discharged to home), despite all patients meeting the Medicare eligibility criteria for hospice in the United States. Of those discharged to hospice, this cohort were older in age, more likely to have ascites and prior infections requiring high volumes of paracentesis, and less likely to be listed for liver transplantation. Grade 3–4 hepatic encephalopathy was also found to be the greatest predictor of discharge to hospice. However, the authors noted that low MELD scores (models for stratifying the severity of end‐stage liver disease for transplant planning) were associated with patients discharged to home, which may not adequately capture debilitating hepatic encephalopathy and/or refractory ascites, leaving them to suffer in what they called ‘MELD purgatory’ where low scores do not reflect patients' symptom burden (p2575). Finally, Fukui et al. (2017) reviewed hospitalised Medicare beneficiaries (United States; aged over 65 years or those with qualified health conditions) with chronic liver disease discharged to hospice over a 4‐year period (*n* = 2179) to determine the costs related to the underutilisation of hospice (as compared to a control of beneficiaries without chronic liver disease). Despite overall consistent yearly increases in discharge to hospice, patients with chronic liver disease had higher rates of hospice death, hospital mortality, and 1‐year mortality compared to those without chronic liver disease. The authors attribute these patterns to higher rates of, and more aggressive, life‐prolonging therapies, in which late referrals to hospice were unlikely to be optimally beneficial.

In interpreting this literature, we found that challenges in ascertaining the end‐of life phase for people with lived experience of HBV or HCV are exacerbated by the conflation of aetiologies into a singular diagnosis of end‐stage liver disease. Without specific mention of hepatitis in the analysis of findings, diversity of experiences leading up to a terminal condition are obscured, and instead collapsed into the various clinical markers for determining end‐of‐life under that disease profile. The response to end‐of‐life status typically results in palliative or hospice referral, with less evidence of the use of other services that can support patients during a period of fluctuating illness, which may or may not be ultimately curative given the potential of liver transplantation. Yet, despite the centrality of referral as proxy of the end‐of‐life stage, people with end stage liver disease are referred late or not at all.

### 3.2 Challenges in ascertaining the range of end‐of‐life care in the context of ageing with chronic viral hepatitis

It is important to interpret findings relating to how support is identified and practiced within the literature scoped in this review in light of the disparity in how end‐of‐life is determined. Over half (*n* = 14) of the articles analysed approached support at the end‐of‐life stage within the domain of specialist palliative care services (and in some cases, discharge to hospice as equivalent). In the scoped literature, we interpreted care broadly as falling under categories related to: i. clinical (prognosis assessment, symptom management, including physical/somatic and/or psychological), ii. educational (need for advance care directives and surrogate decision makers, discussion of treatment options and determining goals of care) and iii. Psychosocial (quality of life beyond symptom management, emotional/spiritual support and family and bereavement support). Educative components (e.g. advance care planning and goals of care discussions) occurred as part of broader clinical care (Baumann et al., 2015; Carbonneau et al., 2018; Chen et al., 2021; Ebenau et al., 2019; Kimbell et al., 2015; Lamba et al., 2012; Poonja et al., 2014; Shinall et al., 2019), or as a separate or specialty service available in specialist palliative care referrals (Low et al., 2017; Najafian et al., 2019; Patel et al., 2020; Sprange et al., 2020; Valery et al., 2017; Wang et al., 2021). Other articles discussed disease treatment more broadly (Hansen, Rosenkranz, et al., 2015), symptom management outside of palliative specialities (Hansen et al., 2014; Hansen, Leo, et al., 2015) and emotional and other support needs (Gray‐Renfrew et al., 2020; Valery et al., 2017) outside of palliative specialities.

Studies overwhelmingly reported the clinical aspects of end‐of‐life care (i.e. prognosis assessment and symptom management). Intervention studies introduced early (Baumann et al., 2015; Shinall et al., 2019) and structured (Lamba et al., 2012) palliative care consultations into standard liver transplantation and inpatient hepatology services, in effect bundling palliative care with ongoing clinical assessment and care. All studies found clear benefits in the concurrent provision of palliative and curative care, documenting improvements in symptomatology, including depressive symptoms (Baumann et al., 2015), and decreases in length of hospital stay (Lamba et al., 2012; Shinall et al., 2019), and increases in withdrawal of life‐support interventions (Lamba et al., 2012) compared to those patients not receiving the intervention. Importantly, the need for effective clinical management is supported by measurements of physical and psychological distress in patients, where higher rates of symptomatology among people living with HCV compared with other aetiologies of end‐stage liver disease were found, specifically fatigue (Hansen, Leo, et al., 2015) and pain burden (Hansen et al., 2014). Rather than treating for presence of pain, Hansen and colleagues (2015) recommend that clinical management include attention to shifts in frequency, severity and distress levels at different points in patients' disease course. Reporting on the same study within a different article, Hansen et al. (2014) also draw attention to the intersection of pain with other symptoms, such as fatigue, and how patients manage pain symptoms on their own, such as napping and reducing activity levels, as well as seeking professional help and taking pain medication, which point to other measures of support outside of clinical interventions. In addition, the authors found that 30%–40% of their sample reported no pain medication use despite reporting moderate pain, but they also noted that clinical pain management differs between those on and those declined/delisted for liver transplantation waiting lists (e.g. strong opioid vs. weak medication). Another study similarly reported that despite support accessed by patients diagnosed with cirrhosis in a liver clinic that included a range of allied health services, such as dieticians, pharmacists, psychologists/psychiatrists, spiritual support and social workers, there was less use of those allied support services compared to use of services related to acute symptomatology (Valery et al., 2017). Finally, while patients' acute medical care is well‐coordinated, there was less attention to ongoing care in the community (Kimbell et al., 2015).

There was mixed evidence in terms of outcomes related to educational aims (i.e. advance care directives and surrogate decision makers, discussion of treatment options, determining goals of care and so on) among this literature. This points to a need for clearer understanding about the role of preparation for end of life within clinical care. In intervention studies that showed clear clinical benefits in early referral to palliative care, Baumman and colleagues (2015) reported no increase in rates of registration of healthcare power of attorneys and advance care directives on patients' medical records, while Lamba et al. (2012) reported increased instances of goals‐of‐care discussions on physician rounds and significant increases of do‐not‐resuscitate statuses registered. Yet, in retrospective analyses of patient records, there were overall low registrations of formal acute resuscitation plans (Chen et al., 2021) and do‐not‐resuscitate documentation (Poonja et al., 2014), which complicate Lamba and colleague's findings outside of specific interventions. While Low et al. (2017) noted that two‐thirds of their purposefully sampled medical records had do‐not‐resuscitate (DNR) directives, they also noted that these were completed by medical personnel with limited evidence of consultation with patients or their families, and most patients (63%) had no discussions about their preferred place of care or death. These findings were similarly reflected in other studies using quantitative measures that reviewed advance care planning in the context of cirrhosis. Among patients undergoing liver transplantation evaluations, Wang et al. (2021) found that although 9% of patients reported completion of advance care planning, none of them had documentation on end‐of‐life preferences or directives in their medical records, and of those with notes on discussions of advance care planning, these were only found in notes by social workers, not transplant hepatologists or surgeons. Najafian et al. (2019) found that despite a structured program bridging inpatient hepatology care to outpatient clinics (a transitional care liver clinic), there was no discussion of advance care planning for patients during visits over the course of 1 year. Similar to Wang and colleagues' findings, Najafian and colleagues reported that 66% of patients had hepatic encephalopathy and 56% were not accompanied by a family member or caregiver, which presents additional challenges in navigating decision‐making and support needs. Following a public awareness campaign, Sprange et al. (2020) likewise found under half of their surveyed patients with cirrhosis had heard of advance care planning, goals of care designation and personal directives, with even fewer reporting that they had a good understanding of the process. These findings were similarly reflected in Valery et al.' (2017) study that found that 80% of their cohort did not know what an advance care directive was, with only 2% having one documented, and in Carbennau and colleagues' (2018) study that found that few participants understood the role of goals of care designations, even if they had one recorded. Uncertainty about disease trajectory was found to be a factor in patients' level of readiness to engage in advance care planning discussions among a variety of patients experiencing liver cirrhosis (Carbonneau et al., 2018).

There was very limited evidence of the uptake of psychosocial dimensions (i.e. quality of life beyond symptom management, including emotional/spiritual support, and family and bereavement support) of care at the end of life across the literature reviewed. A cross‐sectional study found that patients with liver cirrhosis were just as concerned about emotional support and support to cease alcohol and substance use as they were about clinical symptom management and disease management (Valery et al., 2017). Indeed, negative emotional responses to advanced liver disease were equally evident in a secondary analysis of a longitudinal study, with Gray‐Renfrew et al. (2020) describing fear, anger, guilt and shame, with causal factors associated with these emotional responses, including shock of diagnosis, uncertainty around declining health and pessimism over lack of cure, which point to the need for further psychosocial support. Uncertainty was a dominant theme in perspectives of patients recruited from inpatient hepatology wards, evident in patients' confusion between symptoms associated with the disease and those related to chronological ageing, confusion over the term ‘cirrhosis’ and its relation to liver disease, and the unpredictable trajectory of the illness following diagnosis (Kimbell et al., 2015). Uncertainty was also evident in how the disease impacts everyday life, with limited awareness of psychosocial support services available, which was further hampered by a perceived lack of communication, information and education from healthcare professionals. Further, in another study of patients with advanced hepatocellular carcinoma at the end of life (Hansen, Rosenkranz, et al., 2015), uncertainty intersected with illness perception and decision to start and navigate treatments—all of which affect patients' perception of quality of life. Importantly, the authors found that patients' perception of illness and treatment decisions changed over time, and that perception of a greater need for the patient to control how the disease affected quality of life influenced how they navigated treatment decisions. In the provision of palliative services or liver transplantation services, shame and stigma was found to be compounded by the association of the disease with risky or stigmatised lifestyle behaviours (i.e. injecting drug use, alcohol consumption, sex between men and so on) (Gray‐Renfrew et al., 2020; Hansen, Rosenkranz, et al., 2015; Lamba et al., 2012).

These latter findings support the need for psychosocial components of care, including recognition of the potentially complex life circumstances of those living with viral hepatitis, that intersect with clinical and educational needs at the end‐of‐life phase. However, the literature scoped in this review found greater emphasis on the clinical aspects of care, with less attention paid to educative dimensions. This poses challenges when only one point of end‐of‐life and palliative care planning and practice are considered with respect to people with lived experience of HBV or HCV. There is a lack of evidence in advance care planning beyond essential clinical arrangement (such as DNRs) at the very end of their life, which points to missed opportunities for considering educative and psychosocial support needs that could be coordinated by a multi‐disciplinary health and social care team prior to the end‐of‐life phase.

## 4 DISCUSSION

Given improvements in treatments (and cure in the case of HCV) have resulted in more people with lived experience of HBV and HCV now reaching older age, there is a clear need for an evidence base to understand how care and support is provided for this emerging population as they near the end‐of‐life phase. In addition to understanding the effects of HBV and HCV as part of a conventional chronological ageing perspective, further attention is needed to disentangle complex relationships between, on one hand, biological ageing, long‐term treatment effects and chronic hepatitis disease progression, and, on the other hand, social, behavioural and structural impact on this population. In the literature scoped for this review, there was an overwhelming focus on end‐stage liver disease, which obscures all other instances where people living with chronic viral hepatitis may have other health issues actively contributing to morbidity but hepatitis is not directly responsible for death (Podymow et al., 2006). This focus presents challenges in teasing out other experiences of dying in the context of ageing that may be relevant to people with lived experience of HBV or HCV. This scoping review also underscores that palliative care research does not focus on specific conditions, for example, a blood‐borne virus, but rather focusses on outcomes, such as symptom management.

Although scoping reviews do not evaluate the methodological quality of the included research, there are some limitations that affect the generalisability of the research scoped, including: only articles published in English were included, which may have resulted in Western biases towards end‐of‐life definitions and approaches (and indeed, although hepatitis B is prevalent among diverse culturally and linguistically diverse groups globally, this population was underrepresented in the articles scoped); a dominance of studies conducted in the United States, which may not be generalisable to other jurisdictions given differences in particular population, legal and insurance contexts; an emphasis on hepatology and oncology specialist domains in the treatment of liver disease, rather than, say, infectious diseases specialists for viral hepatitis and the majority of results and discussions that did not report data separately on disease aetiologies or bundled disease aetiologies in the presentation of findings, which makes it difficult to determine hepatitis‐related mortality and morbidity as distinct from other liver disease causes. HCV was overwhelmingly represented in the studies under review, and in some cases, HBV was specifically excluded from studies (Fukui et al., 2017) or combined with HCV as ‘viral hepatitis’ (Brown et al., 2016; Sprange et al., 2020). HBV and HCV each have unique epidemiological, clinical and social trajectories. Though not intending to conflate the specific challenges inherent in both HBV and HCV, we undertook this scoping review through the lens of commonalities for end‐of‐life care among people with chronic viral hepatitis as they often have pre‐existing or quickly emerging socio‐economic disadvantages and co‐morbidities. Moreover, as our focus was on end‐of‐life at the intersection of ageing (i.e. not acute progression in younger people), older people were overwhelmingly represented in the articles selected for inclusion, reflected in the higher median or age range of participants included in those scoped studies. However, despite these limitations, our scoping review of the challenges in determining this phase of life, as well as the types of care provided, confirm two key domains that require further consideration.

First, across the literature, clinical indicators that determine an end‐of‐life stage were based on advanced or end‐stage liver disease, though each study had varying inclusion criteria. This implies that there is a demarcation between ageing and dying, at which point clinicians refer to or integrate palliative care. This presents difficulties in determining end‐of‐life in the context of ageing, where this life stage is determined by disease progression rather than comorbidities associated with ageing. Indeed, HBV or HCV may not trigger the conventional palliative care pathway in the absence of end‐stage liver disease. Furthermore, the challenges faced in distinguishing the end‐of‐life phase are not uncommon with other chronic conditions (e.g. heart failure and chronic obstructive pulmonary disease). Yet, variation in determining end‐of‐life has implications for treatment, continuity of care, quality of experience, pain management, preparedness for death and so on, and so understanding this variability in each context and among each population group is important in how care needs are determined. In interpreting these findings, the authorship team took care not to reify specialist palliative care as the only form of end‐of‐life care, as referral to specialist palliative care is only one indicator of awareness regarding the need for tailored care for the dying among other care models. For example, while it is important to determine the end‐of‐life as a phase over a patient's life course, this does not automatically imply the need for end‐of‐life care, either through specialist palliative care referral or care provided by a specialist clinician or general practitioner. A balance may be required between disease and treatment burden to ensure quality of life is maintained (Trevena, 2018). This has other practical implications in terms of identifying the ‘transition’ stage where there is likely to be significant variation related to family history and cultural interpretations. Moreover, cultural perceptions of stigma associated with viral hepatitis infections can intersect with values or understandings of end‐of‐life choices in various cultural contexts.

Second, a prevailing conclusion among these studies was that palliative care was underutilised and referrals to palliative care services were engaged too late for patients with end‐stage or advanced liver disease (Brown et al., 2016; Chen et al., 2021; Ebenau et al., 2019; Fukui et al., 2017; Low et al., 2017; Poonja et al., 2014; Shinall et al., 2019). This was the case for both specialist palliative care, delivered by specialist care teams or in hospice settings, and generalist palliative care delivered by oncologist or other clinicians during the course of clinical management. The research inclusion criteria resulted in an emphasis on clinical care, and a corresponding lack of literature related to psychosocial needs outside of clinical and (to a lesser degree) educative needs related to the end‐of life‐phase. This review also evidences the benefits of early palliative referral and of bundling tertiary and palliative care concurrently, in which intervention data demonstrate improvements in clinical components of palliative care when combined with curative treatment and care (Baumann et al., 2015; Lamba et al., 2012; Shinall et al., 2019). Equally, there was clear evidence that poor patient understanding and low rates of discussion around advance care planning and other directives demonstrate the need for early practitioner‐facilitated discussion and documentation. Symptoms of cognitive impairment intersect with liver functionality scores in determining end‐of‐life support, but the lack of psychosocial care presents challenges for the educational aspects of end‐of‐life, such as the need for advance care planning and legal decisions around dying, especially pertinent in broader discussions with families and/or caregivers. Across the literature, we found that advance care planning tends to be more clinical‐focused, including DNR orders, but less focused on broader end‐of‐life planning (such as legal implications, including guardianship, power of attorney, etc), which require additional expertise beyond clinician skills. While there is more emphasis on the clinical and educative needs and less is known about psychosocial needs, the latter may be more important given the socio‐cultural dimensions and diversity of people with lived experience of chronic viral hepatitis.

Our synthesis of the literature scoped for this review has implications for policy and practice. Different clinical disciplines have different markers of end‐of‐life and dying, but the transition between them is not as clear. There is a compelling need for clinicians to see ageing and dying as on a continuum with intersecting care and support needs, rather than being triaged into separate and distinctive life course stages. Given other aged‐related morbidities, and other care and support needs, there are additional opportunities to consider not only the relationship between palliative care and oncology, but other relationships between other disciplines and specialities because of the intersecting multimorbidity that may be present. Indeed, any emphasis on oncology in understanding end‐of‐life needs for this cohort may be a limitation as not all people living with chronic viral hepatitis and liver disease die of liver cancer as a direct cause. These challenges are further compounded when the uneven trajectory of viral hepatitis is considered. Patients once considered within the end‐of‐life phase may need to be effectively ‘re‐phased’ following treatment or transplantation. As such, policy approaches to palliative and end‐of‐life care need to consider the ‘spectrum and unpredictability’ of underlying disease (Mazzarelli et al., 2018).

Given the focus on the prominent disease presentation (i.e. liver cirrhosis, end‐stage or advanced liver disease) the social and cultural dimensions of these infections have received less attention. While not covered in detail in the literature, stigma and discrimination are key factors in patient experiences as they near end of life, which present significant concerns around health equity. Indeed, the number of proxies to describe identities and lifestyles associated with hepatitis (injecting drug use, sex between men and so on) should remain a concern for policy makers and clinicians alike. Moreover, stigma plays a critical role in influencing people's decision‐making around disclosure and treatment seeking in both infections (Freeland et al., 2022; Jin et al., 2020; Treloar & Rhodes, 2009), which has implications for clinical management. Stigma and discrimination related to hepatitis serostatus has also been found to pervade all aspects of an individual life (Freeland et al., 2022), suggesting that its effects radiate across the whole life course. The social implications of living with a stigmatised disease, then, not only influence diagnosis, disclosure and treatment decisions, but inevitably those associated with the end‐of‐life. Specialist palliative care providers, as well as generalist clinicians and oncologists, should consider additional care and support needs that relate to the psychosocial elements of end‐of‐life. An emphasis on the provision of clinician‐led information (i.e. by general practitioners or liver specialists) may hinder the provision of more comprehensive information, especially those pertaining to psycho‐social needs, required by patients. Previous studies on HBV have shown that most patients will obtain relevant information pertaining to their experience of the disease from their clinician, not through other potentially culturally resonant sources (Hopwood, 2013). Cultural understandings of life and death need to be acknowledged, which in turn could inform how information is framed and relayed. Indeed, the focus of the scoped articles lies in the structured and biomedical response to end‐of‐life, rather than any care and support that can take place outside clinical settings, which can impede our understanding of the needs of people with HBV given their cultural and linguistic diversity on Australia. Finally, care in the community remains an important consideration for end‐of‐life support. A person‐centred care could benefit from a consideration of the unique experiences of people with lived experience of HBV and HCV where ageing and dying lies on a continuum, rather than a single transition point.

## AUTHORS' CONTRIBUTIONS

The initial idea for a scoping review on the topic came from Kerryn Drysdale, Jake Rance, Limin Mao and Carla Treloar. The search strategy was devised by Kerryn Drysdale and Elena Cama. Screening records was undertaken by Elena Cama, Kerryn Drysdale and Limin Mao, and any discrepancies were resolved through discussion with the team. Data from these records were extracted by Elena Cama, while Kerryn Drysdale and Jake Rance undertook the qualitative thematic analysis. Preparation of the manuscript was undertaken by Kerryn Drysdale, with considerable input from all authors.

## FUNDING INFORMATION

This scoping review was funded by the Australian Department of Health. The contributions of the investigator team were supported by the Centre for Social Research in Health, which receives some funding from a range of external agencies.

## CONFLICT OF INTEREST

The authors declare that there is no conflict of interest.

## Data Availability

Data sharing not applicable to this article as no datasets were generated or analysed during the current study.
